# Experiences in anti-tuberculosis treatment in patients with multiple previous treatments and its impact on drug resistant tuberculosis epidemics

**DOI:** 10.3402/gha.v7.24593

**Published:** 2014-08-18

**Authors:** Biao Xu, Qi Zhao, Yi Hu, Ying Shi, Weibing Wang, Vinod K. Diwan

**Affiliations:** 1 Department of Epidemiology, School of Public Health, Fudan University, Shanghai, China; 2 The Department of Tuberculosis Prevention and Control, Ministry of Health, Beijing, China; 3 School of Public Health, Centre for Global Health, Karolinska Institutet, Stockholm, Sweden

**Keywords:** tuberculosis, drug resistance, second-line anti-tuberculosis drugs, treatment history, China

## Abstract

**Background:**

Tuberculosis (TB) patients with a history of multiple anti-TB treatments are the ‘neglected’ group to the free anti-TB treatment policy in China.

**Objective:**

To understand the experiences of TB patients with multiple previous treatments with regard to bacteriological diagnosis and treatment regimens, especially for second-line anti-TB drugs, and how this might influence the risks of multidrug and extensively drug-resistant TB (M/XDR-TB).

**Design:**

A cross-sectional study was conducted in 10 county/district TB clinics in five provinces of China. The study participants were TB patients that had at least two previous treatment episodes that lasted longer than 1 month each. Face-to-face interviews and drug susceptibility testing (DST) were conducted with the consenting participants.

**Results:**

A total of 328 TB patients were recruited. The proportion of multidrug-resistant tuberculosis (MDR-TB) was 58.2% in the 287 DST-confirmed patients. Forty-two percent of the patients did not complete their first treatment course. About 23.8% of the participants had a history of taking second-line drugs, and more than 77.8% of them were treated in county TB dispensaries where only sputum microscopy was applied. Multivariate analysis found that the use of second-line drugs was significantly associated with frequency of previous treatments (*p*<0.01), but not with drug resistance profiles of patients.

**Conclusions:**

Patients with multiple previous treatments are at extremely high risk of MDR-TB in China. The unregulated use of second-line drugs bring about the threat of XDR-TB epidemic. DST-guided treatment and strict regulations of anti-TB treatment should be assured for the high-risk TB patients for the prevention and control of M/XDR-TB.

During the last two decades, as a country having the second highest burden of tuberculosis (TB) worldwide, the Chinese government has made tremendous efforts in the fight against TB (1). The global rise in multidrug-resistant tuberculosis (MDR-TB) and more recently extensively drug-resistant tuberculosis (XDR-TB) stands to thwart decades of progress in TB control. Drug resistance in China has been rapidly growing and becoming a cause for critical public health concern. A recent national survey of drug-resistant TB in China estimated an astounding 5.7% of new cases (incident) and 25.6% of previously treated cases harbored MDR-TB in 2007. Approximately 8% of the patients with MDR-TB had XDR-TB (2). It was estimated that there were 110,000 incident cases of MDR-TB and 8,200 incident cases of XDR-TB among all registered TB patients in 2007 (2). Thus, China harbors the largest absolute number (a quarter) of MDR-TB cases globally and perhaps the largest reservoir of individuals latently infected with M/XDR mycobacteria TB (3, 4).

Although, primary transmission of M/XDR strains contributes in part to the ongoing incidence, it appears that one of the key driving forces behind the rise of M/XDR-TB is poor case management (5, 6). A major component of the directly observed treatment-short course (DOTS) strategy adopted in national TB control programs is the standardized chemotherapeutic treatment with supervision by healthcare providers to ensure patient adherence and treatment completion. However, in practice, drug resistance has been reported in association with TB control programs in virtually all countries. Problems on treatment compliance and appropriate regimens have certainly played a role in the emergence of M/XDR in China.

The basic unit of China's TB control program is the county/district TB dispensary where patients from the administrative area receive diagnosis, treatment and management. Patients are primarily diagnosed by smear microscopy examination, and if indicated, are given a first-line anti-TB drug-based 6-month chemotherapy for new TB cases and 8-month therapy for retreated cases free of charge. Mycobacterial culturing and drug susceptibility testing (DST) are not routinely performed due to shortage of facilities and human resources. Standardized regimentation of second-line anti-TB drugs has not been introduced to treat the MDR-TB patients in county and district level, but fluoroquinolones (FQ) and some other injectable second–line drugs have been widely used in general medical practice as a broad-spectrum antibiotic.

At present, China's MDR-TB control program is set in prefectural level, which is the administrative level above counties and districts, mainly in the prefectural designated hospitals. TB patients at high risk of MDR should be referred from county TB clinics to the prefectural hospitals for DST-based diagnosis and MDR-TB treatment. Previously treated TB patients are at the highest risk of drug resistant TB. Since TB patients with multiple episodes of treatment do not meet the eligibility for free treatment in China, and there are no standardization of TB medical services for retreatment failed patients, this population become a ‘neglected’ group, although from the perspective of public health this group could be a long-standing source of the spread of M/XDR-TB. The present study was carried out to understand what patients with multiple previous treatments had experienced on their TB medical care with regard to bacteriologic diagnosis, treatment regimen especially for second-line anti-TB drugs, and how this might influence the risks of multidrug and extensively drug-resistant TB (M/XDR-TB).

## Methods

### Study setting

This is a population-based cross-sectional study carried out in five provinces in middle, east, west, south and north China. Considering bacteria culture and DST were not routinely available in county/district level TB dispensaries, one prefectural city in each province was purposively selected. The selected cities should be covered by the MDR-TB control program (in most case, a Global Fund to Fight AIDS, TB and Malaria supported project), which could provide quality assured TB bacteriological diagnosis and treatment to all detected drug-resistant patients from this study. Under each selected prefectural city, a county and a district of all the counties and districts under the administration were selected as the study sites using simple random sampling. In total, 10 counties/districts in five prefectural cities were selected.

### Study subjects

Subjects of the study were active pulmonary TB patients who had experienced at least two anti-TB treatment courses longer than 1 month previously. With the assumption of MDR-TB prevalence as 17.1% in previously treated cases (7), and a precision of ±5% for the 95% confidence interval (CI) (*α*=0.05, *d*=0.05), the estimated sample size was 226 cases.

### Data collection

Eligible study subjects were identified from the patient registry system in the sampled county/district TB surveillance system during April–November, 2011 and were invited for participation consecutively. All invited patients consented to being interviewed during their treatment course by physicians in the TB dispensaries. A structured questionnaire was used which covered general demographic and socio-economic characteristics of patients, and their treatment experiences from the first TB diagnosis to current episode. Medical charts and billing information (if available) from previous treatments were reviewed as supporting documents. Bacteriological confirmation of TB diagnosis and DST for current episode was completed in the laboratory of prefectural TB hospitals. MDR-TB was defined as M. TB isolate resistant to at least isoniazid and rifampin. Other drug–resistant TB was referred to any other combination of drug resistance.

### Data analysis

The database was created through EpiData 3.1 and double entry was ensured. Statistical analysis was carried out by SPSS software 11.5 (Statistical Package for the Social Sciences Inc., Chicago, IL, USA). The χ^2^ test was used to assess variations in the proportion of anti-TB drug use between the groups with different socio-demographic and clinic characteristics. The odds ratio (OR) and 95% CI were calculated to investigate factors influencing the use of second-line drug among the subjects. *P*<0.05 was considered as statistically significant.

### Ethical consideration

The Institutional Review Board of Fudan School of Public Health approved the study. Written informed consent was obtained from all participants.

## Result

### General characteristics and TB history of subjects

A total of 328 eligible TB patients were recruited continuously during the study period, and 91.2% were sputum smear positive. The distribution of patients in the five provinces were relatively even from 60 to 81. The mean age of patients was 48 years with 25.2% being above 60 years and a male to female ratio 1:0.41. Of the 328 participating patients, there were 256 patients (78%) who received the first TB diagnosis after 2001 when the DOTS strategy has been adopted widely in the country. About 70.4% (231) of the patients had two longer than 1-month anti-TB treatment episodes previous to the current treatment, and the other 97 patients had experienced 3–5 treatment episodes.

### TB history and drug resistant status

All the recruited patients were referred to prefectural TB hospitals for bacteriology diagnosis, and 300 completed the diagnosis. *M.TB* complex were recovered from 289 patients, another five strains were *non-tuberculosis mycobacteria* (NTM), and the remaining six were negative in bacteria culturing. DST for first-line anti-TB drugs was completed in 287 *M.TB* isolates. The prevalence for resistance to a single drug, multidrug-resistant and other combinations of drug resistant were 17.07% (95% CI: 13.16~21.85%), 58.19% (95% CI: 52.76~64.09%) and 8.36% (95% CI: 5.68~12.14%) respectively and there were only 47 patients (16.4%) who were susceptible to all the first-line drugs (Table 1).

**Table 1:** Prevalence of drug resistance to first-line anti-TB drugs (n=287)

Susceptibilities	Anti-TB drugs	No	%	95% CI
Susceptibility to all four first-line drugs		47	16.40	12.55–21.10
Resistant to a Single drug	Rifampicin	9	3.14	1.66–5.86
	Isoniazid	16	5.57	3.46–8.86
	Streptomycin	18	6.27	4.00–9.69
	Ethambutol	6	2.09	0.96–4.48
Multidrug-resistance (MDR)	Rifampicin+Isoniazid	34	11.85	8.61–16.10
	Rifampicin+Isoniazid+streptomycin	57	19.86	15.66–24.86
	Rifampicin+Isoniazid+ethambutol	15	5.23	3.20–8.45
	Rifampicin+Isoniazid+streptomycin+ethambutol	61	21.25	16.91–26.35
Other drug resistance	Streptomycin+rifampicin	11	3.83	2.15–6.73
	Rifampicin+ethambutol	7	2.44	1.19–4.95
	Isoniazid+ethambutol	1	0.35	0.60–1.95
	Streptomycin+ethambutol	1	0.35	0.60–1.95
	Rifampicin+streptomycin+ethambutol	1	0.35	0.60–1.95
	Isoniazid+streptomycin+ethambutol	3	1.05	0.36–3.03

### Treatment experiences and uses of second-line drugs

More than 60% of the patients were treated in the county/district TB dispensaries for their first and second treatment episode and 57.2 and 54.7% of them completed the treatment course, respectively. About 71.2% of the patients had received the standardized anti-TB therapy in their first treatment episode, and 55.0% in their second episode. The others were treated using loose drugs or other combinations (Table 2). For the 256 patients diagnosed after 2001, the proportion of standardized treatment in the first and second treatment was 76.1 and 55.9%, respectively, and the treatment completion was 49.0 and 37.5%, respectively.

**Table 2:** Treatment experiences of patients with multiple previous treatments

	Episode 1		Episode 2		Episode 3		Episode 4		Episode 5	
	
Treatment	No.	%	No.	%	No.	%	No.	%	No.	%
No. of patients	328	–	328	–	97	–	14	–	5	–
Treated in TB dispensary	207	63.1	220	67.1	39	40.2	5	26.3	1	20.0
Treatment completed	189	57.6	179	54.7	38	40.2	7	50.0	3	60.0
Treatment regimen*										
2HRZE/4HR for new cases‡	233	71.2	58	17.7	9	9.3	1	7.1		
2HRZSE/6HRE for retreat cases‡	8	2.4	180	55.0	38	39.2	2	14.3	1	20.0
6ZKmLfxPtoPAS/18ZLfxPtoPAS‡			6	1.8	3	3.1	1	7.1		
Single drug: INH/RMP/STR	2	0.6			1	1.0				
Loose drugs§	85	26.0	84	25.6	46	47.4	10	71.4	4	80.0

* Definition of anti-TB drugs: First-line anti-TB drugs refers to the most important drugs for the treatment of TB including isoniazid (H), rifampicin (R), pyrazinamide (Z), ethambutol (E) and streptomycin (S). Recommended treatment regimen for new TB case, retreated TB case and MDR-TB case. Second-line anti TB-drugs: Second-line anti TB drugs investigated in this study included para-aminosalicylic acid (PAS), kanamycin (KM), amikacin (AMK), Capreomycin (CPM), ofloxacin (OFX), levofloxacin (LVX), ciprofloxacin (CFX), prothionamide (PTH) and cycloserine (CS).

‡ Recommended treatment regimen for new TB case, retreated TB case and MDR-TB case.

§ Combination unclear.

It was found that 66 of the 328 patients (23.8%) had a history of taking second-line drugs, of which, 5.5 and 14.6% occurred in their first and second treatment episode respectively; of these patients, 77.8 and 39.6% respectively were treated in county/district TB dispensaries where only sputum microscopy was applied in diagnosis (Table 3). The most frequently used second-line drug was FQs, and 62.5% had used two or more second-line drugs in a single treatment episode. Second-line drugs were used mainly as the supplements to the recommended regimen in 54 of the 66 patients.

**Table 3:** Experiences of patients using second-line anti-TB drugs in previous treatments

		No. of patients using second-line drugs					
		
		No. of drugs (%)			Distributions of health facilities		
		
Episode	No. of patients	Total	1 drug	>1 drug	CTD	Hospital	Others
1	328	18 (5.5)	9 (50.0)	9 (50.0)	14 (77.8)	4 (22.2)	–
2	328	48 (14.6)	25 (52.1)	23 (47.9)	19 (39.6)	28 (58.4)	1 (2.1)
3	97	44 (45.4)	12 (27.3)	32 (72.7)	4 (9.1)	36 (81.8)	4 (9.1)
4	19	9 (47.7)	1 (11.1)	8 (98.9)	–	6 (66.7)	3 (33.3)
5	5	2 (40.0)	–	2 (100.0)	–	2 (100.0)	–

The proportions of having used second-line drugs in currently susceptible, MDR and other drug-resistant TB patients were 14.9, 19.5 and 13.3%, respectively without any statistically significant difference (*χ* ^2^=1.342, *p*=0.511) (Table 4).

**Table 4:** Using of second-line drugs* in patients with different profiles of first-line drug susceptibility

	Second-line drugs not used		Second-line drugs used	
	
Susceptibility/resistance	No.	%	No.	%
Susceptible	40	85.1	7	14.9
MDR-TB	120	80.5	29	19.5
Other resistance	52	86.7	8	13.3
Total	212	82.8	44	17.2

*χ* ^2^=1.342, *p*=0.511.

* Missing value: 21.

Multivariate analysis was applied to understand the association between the use of second-line drugs and patients’ demographics, socio-economic status, and drug resistant profiles. It was found that frequency of previous treatment episode was significantly associated with the use of second-line drugs. Compared to patients with only two previous treatments, those with three episodes (OR=4.587, *p*<0.001) and four episodes (OR=16.178, *p*<0.001) had a significantly higher possibility of using second-line drugs. No statistically significant association was found between MDR status and the use of second-line drugs in these patients (OR=0.907, *p*=0.873); neither were other drug resistance, age, gender and medical insurance status of patients (Table 5).

**Table 5:** Factors influencing using second-line anti-TB drugs by logistic regression analysis

				95% CI	
				
Factors	*β*	*p*	OR	Lower	Upper
Gender (M vs. F)	−0.619	0.160	0.539	0.227	1.276
Age (years)	−0.023	0.145	0.977	0.946	1.008
Average annual income (CNY)	0.157	0.501	1.170	0.741	1.848
No. of treatment episode		0.000*			
3 vs. 2	1.529	0.001*	4.615	1.860	11.446
4 vs. 2	2.814	0.000*	16.683	3.510	79.302
Education years (>6 vs. ≤6)	−0.897	0.071	0.408	0.154	1.079
Medical insurance		0.012			
NCMS vs. none	−0.867	0.188	0.420	0.116	1.526
Others vs. none	0.746	0.263	2.109	0.571	7.790
Smear microscopy (+ vs. −)	0.276	0.763	1.318	0.219	7.932
DST of first-line drugs		0.947			
MDR vs. Susceptible	−0.222	0.870	0.905	0.273	2.997
Other DR vs. Susceptible	−0.123	0.752	0.801	0.201	3.187

* : p<0.05 and NCMS: New collective medical scheme.

## Discussion

It is striking that the proportion of MDR-TB was 58.2% in patients with multiple previous treatments. About 5.5 and 14.6% of the participating patients in this study had taken second-line drugs in their first and second treatment episode respectively, and the majority of them were treated in the basic TB care unit where only sputum microscopy was applied in diagnosis. Multivariate analysis found that there was no statistically significant association between the use of second-line drugs and the drug resistant profiles of patients.

### Treatment management and risk of MDR-TB

The current global crisis of drug-resistant TB is due to a combination of both acquired resistance and primary transmission (8, 9) MDR-TB, and the even severe form of drug resistance, XDR-TB, is a man-made problem. It is based on the stepwise chromosomal mutations/selections during sub-optimal exposure to TB drugs. So MDR-TB comes about as a result of the combined mutations in several genes and their interaction with each other and with a range of environmental factors (3.4). A number of socio-demographic, clinical and health system factors including previous treatment mismanagement, lack of direct observation (DOT), poor compliance with anti-TB therapy, decreased patient immunity, low case detection and poor infection control have been reported being associated with M/XDR-TB (10).

Patients having previous anti-TB treatments are at the highest risk of MDR-TB (2, 5, 11, 12). China's drug resistant TB survey reported that the relative risk (RR) for MDR occurrence was 2.8 and 7.0 times higher in patients having two or more than two previous treatment episodes compared to new patients (2). In our study, of the 328 patients with multiple previous treatments from five geographically diverse provinces, the prevalence of MDR-TB was as high as 58.19%.

When TB patients are treated in carefully controlled settings, the emergence of drug resistance is rare. Globally, the national TB control program has specifically emphasized the standardization of TB treatment in terms of regimen, course and the DOT. But in this study, it was found that more than 40% of our patients did not complete their first treatment course and about one-fourth of the patients did not receive the recommended standardized regimen although 63% received their first treatment course in the designated TB dispensaries. Even for the 256 patients diagnosed after 2001, the proportions of treatment completion and standardized regimen taken were only 49 and 76.1% for patients at their first treatment episode. Findings from this study implied that irregular treatment happened in Chinese patients, even when the DOTS program has been widely adopted. A study in one province of China found that 12% of patients did not take at least 10% of their prescribed doses of anti-TB treatment (13). Also, nearly 50% of previously treated MDR-TB patients identified in China's survey of drug resistant TB had not completed their last regimens (2). Several other studies showed a low treatment adherence among sputum smear-positive TB patients in China (14–16). A study in a mountainous area reported that only about 29% of the 524 TB patients took drugs under direct observation during the intensive phase of treatment (17). Another study carried out in Chongqing, one of the most popular cities, showed that only 16% of studied patients reported being directly observed whenever they took medicine; less than 5% were observed by health staff and direct observation was neither well understood nor thought to be necessary (18). DOT is helpful considering the length of TB treatment and the potential side effects of the drugs. However, accomplishing qualified DOT by health providers is a challenge especially in countries with high TB burden, poor resources and weak capacity in TB health care (19, 20).

### Needs for drug susceptibility-guided treatment

Despite the increasing prevalence of drug-resistant TB, most low- and middle-income countries use standardized regimens without assessment of drug susceptibility. In this study, none of the patients reported they had received DST before; and more than 60–70% patients underwent their first and second treatment course in the basic TB control unit where infrastructure, human resource and financial input could hardly meet the requirements for bacteriological-based TB diagnosis in terms of biosafety, accuracy and cost.

Findings from a systematic review showed that a relapse of TB was most strongly associated with initial drug resistance (21). The increasing risk of MDR in newly diagnosed TB patients underscores the urgent needs for DST in basic units of TB control in high TB burden countries (22, 23). The initiation of treatment and the choice of chemotherapy regimens should be based on the results of DST. This could be achieved through greater use of existing technologies for drug-sensitivity testing or through the adoption of recently developed rapid tests for drug resistance (24, 25). Considering TB is prevalent in poor population and areas, cheap, maneuverable, safe and rapid diagnosis tools of drug resistance should be established in the basic TB health facilities.

### Use of second-line anti-TB drugs and risk of XDR-TB

Treatment of drug resistant TB requires using the second-line drugs. For the prevention of resistance to the second-line anti-TB drugs, especially for the XDR-TB prevention, prudent use of drugs, treatment follow-up, supervision, and case management should be regulated (26). But irrational use or non-DST-guided use of second-line drugs in TB treatment have been observed in China. A nationwide study carried out in 4,782 health facilities (1960 involved in TB medical care) in 12 provinces reported that 41.8% of the county-level TB clinics and designated hospitals had at least one second-line drug available, and none of them were able to carry out the DST. In the county-level TB dispensaries/clinics, the availability of any second-line drugs, AMK and FQ were 34.4, 9.8 and 26.1%, respectively (27).

To date, there are no published data on the use of second-line drugs in the routine practice of TB treatment in China. In this study, apart from the fact that 23.8% of our patients had a history of taking second-line drugs, it is worse to see that there was no significant association between the use of second-line drugs and drug resistant status of patients, which implied that doctors prescribed the second-line drugs based on clinical judgments. Studies in China have reported that baseline resistance to second-line drugs has become common, for example, the prevalence of OFX and AMK resistance was respectively 13 and 7% in new patients, 27 and 21% in retreatment patients, and 48% in MDR-TB patients (28–30). For the prevention and control of XDR-TB epidemic, strict regulations of second-line drug prescription, monitoring of second-line drug use should be strengthened and implementation of quality assured DST needs to be scaled up urgently in China's national TB control program.

### Limitations

Although the study was carried out in 10 counties/districts in five geographic varied provinces of China, it might not be a good representative of China's more than 2,800 counties. Also, due to the constrain in DST availability, subjects recruited in this study were those who had been recommended to up-level prefectural TB hospitals for drug resistance tests and further treatment under the MDR-TB control project, thus the prevalence of MDR-TB and incompletion of initial and retreat treatment courses might be overestimated. However, this was the only ethically acceptable way to carry out the survey in this neglected population since under the MDR-TB control project the bacteriologic diagnosis and further treatment could be assured. Although with the expectable intervention, the consecutive enrollment of the patients with multiple episodes of treatment could, to a large extent, reflect the true situation in the local settings to some degree. Another limitation of this study was that the billing information was not always available. Lack of electronic records or detailed chart records on doses, durations and other information of non-recommended regimen made it difficult to understand the reasons and driving factors in the selection of treatment regimen and the extents of irrational uses of second-line drugs.

## Conclusion

The worst situation of MDR-TB is observed in patients with multiple previous treatments. These patients in rural China had experienced treatment failures mainly in the low-level TB health facilities. Second-line anti-TB drugs have been used in new and retreated TB patients in TB clinics where drug susceptibility tests were not available. The prescription of the second-line drugs mainly depends on the treatment history of patients, rather than drug-resistance profile. Managing the treatment of existing cases appropriately is the key in prevention of the spread of M/XDR-TB. Strict regulations of anti-TB treatment, monitoring of second-line drug use should be strengthened and implementation of quality assured DST must be scaled up in China's national TB control program.
